# Demographic analysis reveals gradual senescence in the flatworm *Macrostomum lignano*

**DOI:** 10.1186/1742-9994-6-15

**Published:** 2009-07-30

**Authors:** Stijn Mouton, Maxime Willems, Patricia Back, Bart P Braeckman, Gaetan Borgonie

**Affiliations:** 1 Nematology Unit, Department of Biology, Ghent University, Ledeganckstraat 35, 9000 Ghent, Belgium; 2 Research Group for Aging Physiology and Molecular Evolution, Department of Biology, Ghent University, Ledeganckstraat 35, 9000 Ghent, Belgium

## Abstract

Free-living flatworms ("Turbellaria") are appropriate model organisms to gain better insight into the role of stem cells in ageing and rejuvenation. Ageing research in flatworms is, however, still scarce. This is partly due to culture difficulties and the lack of a complete set of demographic data, including parameters such as median lifespan and age-specific mortality rate. In this paper, we report on the first flatworm survival analysis. We used the species *Macrostomum lignano*, which is an emerging model for studying the reciprocal influence between stem cells, ageing and rejuvenation. This species has a median lifespan of 205 ± 13 days (average ± standard deviation [SD]) and a 90^th ^percentile lifespan of 373 ± 32 days. The maximum lifespan, however, is more than 745 days, and the average survival curve is characterised by a long tail because a small number of individuals lives twice as long as 90% of the population. Similar to earlier observations in a wide range of animals, in *M*. *lignano *the age-specific mortality rate increases exponentially, but levels off at the oldest ages. To compare the senescence of *M. lignano *with that of other ageing models, we determined the mortality rate doubling time, which is 0.20 ± 0.02 years. As a result, we can conclude that *M. lignano *shows gradual senescence at a rate similar to the vertebrate ageing models *Rattus norvegicus *and *Mus musculus*. We argue that *M. lignano *is a suitable model for ageing and rejuvenation research, and especially for the role of stem cells in these processes, due to its accessible stem cell system and regeneration capacity, and the possibility of combining stem cell studies with demographic analyses.

## Findings

Flatworms have been an object of ageing studies since Child's initial investigations [1,2]. Researchers tended to focus on the role of stem cells and cell renewal during ageing, and the causal effect of regeneration and starvation on rejuvenation [2-4]. Despite these fascinating themes, the extent of flatworm ageing research remained limited in comparison to that of other model organisms such as *Caenorhabditis elegans*, *Drosophila melanogaster *and rodents. The lack of detailed demographic data partly accounts for this, as the only available data are the maximum lifespans of several species. These data, however, include many discrepancies due to non-specified or non-standardised culture conditions or culture problems such as the presence of fungal and bacterial contaminations [3,4]. Without a basic set of demographic data, the most fundamental question – at which age can an individual be considered old? – remains unanswered. As a result, it is hard to draw any conclusions about, for example, old-age regeneration capacity or the rate of cell renewal as a function of age. Previously published data about these issues are often contradictory or ambiguous and there is still little known about senescence, rejuvenation and the causes of death in flatworms [3,4]. This demonstrates that establishing a survival curve, median lifespan and 90^th ^percentile lifespan is a prerequisite for the experimental design of ageing studies and should be the first step in initiating ageing research with a new model organism. Emerging ageing models are often first described demographically, after which detailed studies follow, stemming from these initial descriptions [5,6]. Because lifespan parameters indicate when individuals can be considered young or old, they allow for choosing age groups to study biomarkers as a function of age and for experiments in which young and old worms are studied comparatively. Furthermore, the survival curve indicates what proportion of the initial cohort is alive at a certain age. Therefore, it can be used to calculate how large an initial culture set-up is needed to retain individuals at a desired age to give the experiment enough statistical power. Besides lifespan parameters, data about the age-related changes in mortality rate provide a basic measure for the rate of senescence [7], and can be used to study rejuvenation by experimental manipulation, such as regeneration and caloric restriction.

In this manuscript, the first flatworm survival curve and demographic dataset are presented. We used *Macrostomum lignano *(Rhabditophora, Platyhelminthes), which is a new model for stem cell biology, development, regeneration and the study of sexual selection [8-13], as well as an emerging model for ageing and rejuvenation research, and especially for the role of stem cells in these processes [14]. Egger et al. suggested that, in *M. lignano*, repeated regeneration induces a lifespan extension and possible rejuvenation [8,9,11], because individuals were cut up to 59 times over a period of 26.5 months and were still able to regenerate [14]. The demographic data in this manuscript can be used to design experiments in which mortality rate parameters are used to conclude whether repeated regeneration slows down the ageing process or induces an actual rejuvenation in comparison to uncut individuals of *M. lignano*.

**Figure 1:**
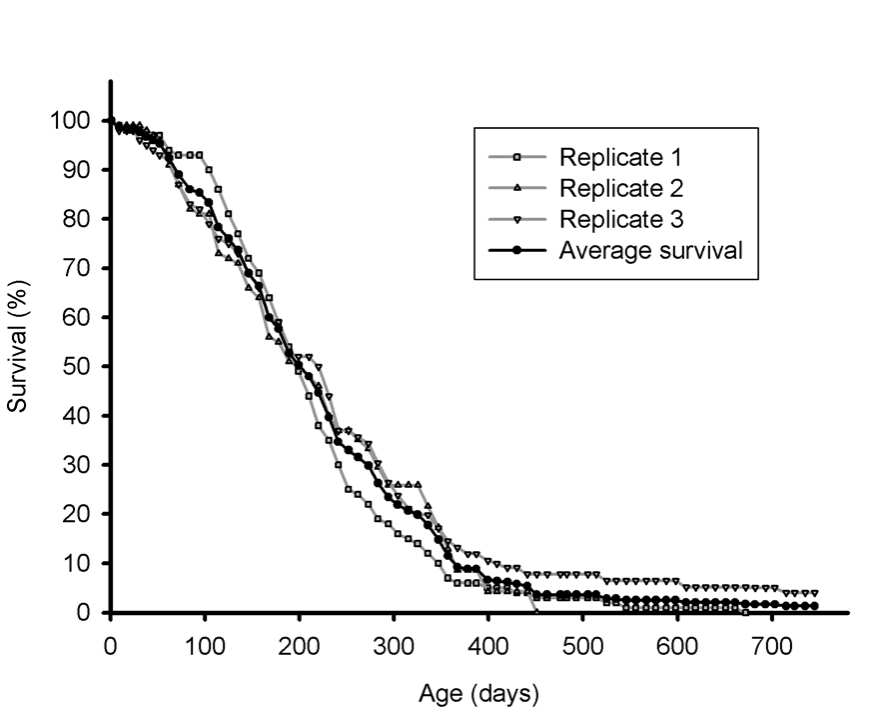
**Survival curve of *Macrostomum lignano***. The grey curves are the survival curves of the separate replicate cohorts. The black, bold curve represents the average overall survival curve of the three replicate cohorts.

**Figure 2:**
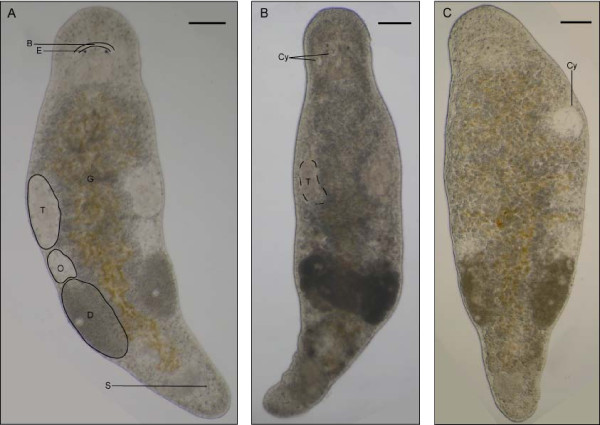
**Morphology as a function of age**. Individuals at four weeks (A), 27 weeks (B) and 76 weeks (C) of age. The brain, left testis, left ovarium and a developing egg are outlined in (A). Furthermore, the organs that can be easily observed are named. B: brain, E: eye, G: gut, which is filled with yellowish diatoms, T: testis, O: ovarium, D: developing egg, S: copulatory stylet. With advancing age, internal organs become less distinguishable as shown for the left testis as an example (A: full line; B: dotted line; C: no line). Furthermore, the body becomes more opaque as a function of age. The opaqueness is, however, variable between individuals and in this figure, it can be best observed in (B). Another characteristic change is the appearance of bulges and grooves in the epidermis. As a result, the right eye is out of focus in (C). The occurrence of body deformities such as cysts (Cy) is also frequently observed. Scalebars: 100 μm.

The data presented here were obtained in three replicate cohorts, maintained under controlled culture conditions. In *M. lignano*, the average (± SD) median lifespan is 205 ± 13 days, the 90^th ^percentile lifespan 373 ± 32 days, and the maximum lifespan more than 745 days (2.04 years). While all individuals in replicate 1 and 2 were already dead at this age, 4% of the individuals in replicate 3 are still alive (figure 1). The lifespan data of the separate replicates are listed in table 1, and individuals of four, 27 and 76 weeks old are presented in figure 2 to illustrate the morphological changes as a function of age. The maximum lifespan here observed is considerably longer than 42 weeks (0.8 year), which was previously reported by Egger et al. [8]. These authors noted, however, that culture limitations like bacteria or parasites may have shortened the lifespan of the specimens. It is remarkable that the maximum lifespan of 745 days is twice as long as the 90^th ^percentile lifespan, which is visualised by the long tail in the survival curve (figure 1), and more than three times the median lifespan, indicating a levelling off of the mortality rate acceleration at advanced ages (see below). This phenomenon was previously described in large medfly cohorts by Carey et al. [5]. Furthermore, these authors concluded that medflies appear not to have a characteristic lifespan, because they observed different maximum lifespans in the several cohorts. Because the maximum lifespan is based on only one individual in the cohort, it results in a great variability which can also be observed in *M. lignano *(table 1). Therefore it is better to use more reliable statistics such as the median lifespan and 90^th ^percentile when demographic data are compared among laboratories, or in experiments studying lifespan extension and rejuvenation.

**Table 1:** Lifespan data of *Macrostomum lignano *in three replicate cohorts (1–3).

Replicate	N(censors)	Median lifespan (days)	90th percentile (days)	Maximum lifespan (days)
1	100 (0)	197	347	672
2	100 (25)	199	362	451
3	100 (9)	220	409	more than 745

All replicates initially started with 100 individuals (N). Individuals that died due to age-independent infection were censored. Median lifespan and 90^th ^percentile are the ages at which 50% and 90% mortality are reached respectively, while the maximum lifespan is the age at which the last survivor of the cohorts dies. At the day of submission the last 4% survivors of replicate 3 were 745 days old, and therefore the maximum lifespan of replicate 3 could not yet be determined.

**Figure 3:**
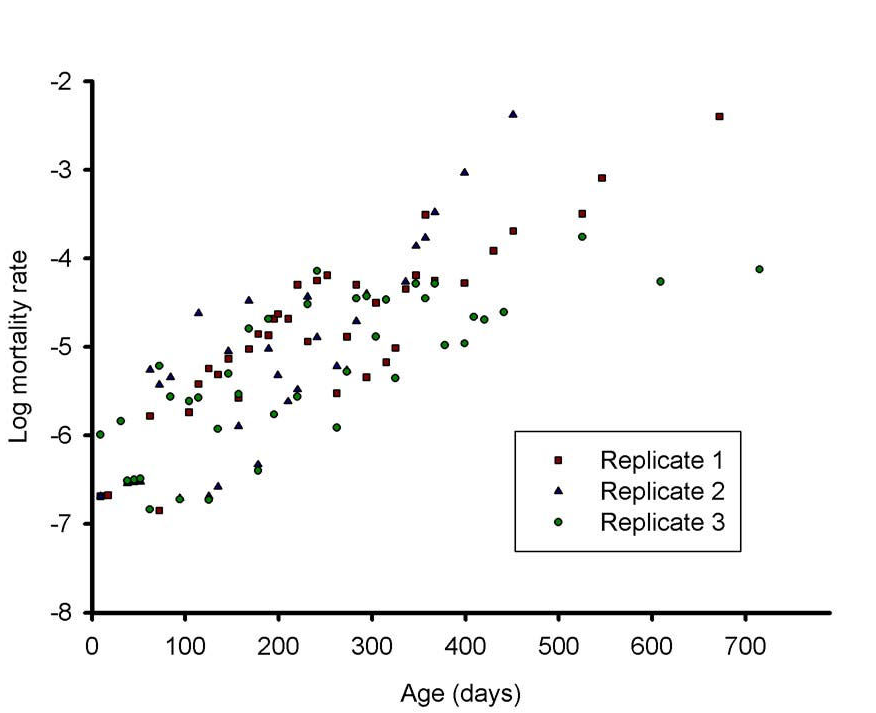
**The logarithm of the mortality rate graphed as a function of age**. Data points represent the age-specific mortality rate of the three replicates. The age-specific mortality rate increases exponentially until the age of approximately one year, after which the increase decelerates. The rate of deceleration varies between the 3 replicates and is especially obvious in replicate 3. Note that data points of age-classes in which no deaths occur are considered as missing because the log (mortality) of these zero-mortality age classes is undefined. This results in large time intervals without data points after the age of one year old, which also reflects the deceleration of the increase in mortality rate.

Lifespan itself, however, cannot indicate the characteristics of senescence, and therefore age-related mortality rate acceleration should be studied in addition. In a wide range of animal populations, including human, an exponential increase in the age-specific mortality rate is observed after maturation and is considered a hallmark of senescence. However, the increase in mortality rate decelerates at advanced ages in several species [5,15]. A similar mortality pattern can be observed in *M. lignano *(figure 3). There are several possible explanations for this, the first one being the genetic heterogeneity of the population. As the population ages, frailer individuals with higher death rates will die out in greater numbers than those with lower death rates, thereby transforming the population into one consisting mostly of individuals with low death rates [5,15,16]. A second explanation is that the chance of death for individuals actually levels off at older ages, resulting in increasing life expectancies at the oldest ages [5,15,16].

To compare the rate of senescence of *M. lignano *with that of other ageing models, the Gompertz function was plotted (table 2). The Gompertz function describes the exponential increase in the age-specific mortality rate, and is the most common model used for interspecies comparison [17-19]. It has proven to be especially useful in simply but accurately describing the kinetics of ageing of populations [20]. Furthermore, the Gompertz coefficient G allows for calculating the mortality rate doubling time (MRDT), which is held to be a fundamental measure of senescence [7,20]. Based on the MRDT, Finch [7] characterised senescence using a continuum with three general subdivisions according to the rate of degenerative changes: rapid (0.005–0.10 years), gradual (0.10–more than 10 years), and negligible (no indication of senescence). Humans, for example, show gradual senescence, with a MRDT of 8 years [7] and maximum lifespan of 122 years [21]. *M. lignano *has a MRDT of 0.20 ± 0.02 years and is therefore also a gradually ageing species. This is in contrast to the commonly used invertebrate ageing models, *Caenorhabditis elegans *and *Drosphila melanogaster*. Both show rapid senescence, with a MRDT of 0.02–0.04 years and a short maximum lifespan of 0.16 and 0.30 years respectively [7]. The vertebrate models *Mus musculus *and *Rattus norvegicus *show gradual senescence, with MRDTs of 0.27 and 0.30 years respectively [7,20]. The MRDT of *M. lignano *is thus much more similar to the rodent models than the invertebrate models. Although *M. musculus *and *R. norvegicus *have a similar rate of senescence, their lifespan is longer: 4.5 and 5.5 years respectively [20]. The median lifespan ranges from about 20 to 30 months in *M. musculus *[22] and is around 30 months for several strains of *R. norvegicus *[23], while it is 205 ± 13 days (between 6 and 7 months) in *M. lignano*. This is due to a larger initial mortality rate (A_0_) in *M. lignano *(average ± SD: 0.42 ± 0.06/year) than in the lab mouse and rat (0.03 and 0.02/year, respectively) [20]. However, we cannot exclude that this initial mortality rate may slightly decrease when culture conditions could be further optimised in this young model.

**Table 2:** Parameters of the Gompertz fit.

Replicate	A_0 _(/day) *(95% CI)*	G (/day) *(95% CI)*	MRDT (years)
1	0.0010 *(0.0006 – 0.0016)*	0.0110 *(0.0089 – 0.0136)*	0.1725
2	0.0013 *(0.0008 – 0.0021)*	0.0087 *(0.0067 – 0.0112)*	0.2183
3	0.0012 *(0.0007 – 0.0020)*	0.0097 *(0.0075 – 0.0125)*	0.1961
Average	0.0011	0.0098	0.1956
SD	0.0002	0.0012	0.0229

A_0 _is the initial mortality rate, which is age-independent. G is the Gompertz coefficient, which expresses the exponential increase in age-specific mortality rate. MRDT is the mortality rate doubling time, which is expressed in years to facilitate comparison with other species. 95% CI is the 95% confidence interval of the parameter as obtained by WinModest. For every parameter, the average and standard deviation (SD) of the three replicate cohorts are calculated and presented.

Studying the influence of treatment on the rate of senescence, e.g. induction of rejuvenation through regeneration, is possible by comparing mortality parameters among treatment replicates [17]. Mortality parameters such the s parameter, describing the degree of deceleration in mortality at older ages, and the age-independent Makeham constant are however not included in the Gompertz function [17]. Therefore, one should first determine the mortality model which best describes the data. Analysis of the data obtained here demonstrates that the average overall survival curve and replicate 1 and 3 are best described by the logistic function, which contains the s parameter, while replicate 2 is best described by the Gompertz function (table 3 and 4).

**Table 3:** Parameters of the Logistic fit.

Replicate	A (/day) *(95% CI)*	B (/day) *(95% CI)*	S (/day) *(95% CI)*
1	0.0003 *(0.0001 – 0.0009)*	0.0260 *(0.0162 – 0.0418)*	2.3416 *(1.1507 – 4.7650)*
2	0.0014 *(0.0010 – 0.0020)*	0.0068 *(0.0054 – 0.0085)*	0.0000 *(0.0000 – 0.0000)*
3	0.0009 *(0.0004 – 0.0020)*	0.0145 *(0.0079 – 0.0266)*	1.3782 *(0.5170 – 3.6739)*
Average	0.0006 *(0.0002 – 0.0016)*	0.0186 *(0.0103 – 0.0333)*	1.7681 *(0.7049 – 4.4350)*

A is the age-independent initial mortality rate, B the acceleration of age-specific mortality rate and S the degree of deceleration of mortality rate at advanced ages. These parameters are given for the three separate replicates and the average overall survival curve. Note that in replicate 2, the S value of zero indicates that there is no deceleration of mortality rate, reducing the logistic model to the Gompertz model. 95% CI is the 95% confidence interval of the parameter as obtained by WinModest.

**Table 4:** Likelihood ratio test.

Replicate	Gompertz	Logistic	P
1	-385.5	-369.9	< 0.001
2	-380.1	-380.1	0.88
3	-378.5	-372.5	< 0.001
Average	-387.4	-378.2	< 0.001

For the three separate replicates and the average dataset, the log-likelihood for the data under the Gompertz and Logistic model are presented. In the last column, the P values of the likelihood ratio tests are presented. The highly significant values in replicate 1, 3 and the average demonstrate that these datasets are best described by the logistic model. The non-significant value in replicate 2 demonstrates that this dataset is best described by the Gompertz model.

The short lifespan in comparison to other gradually ageing models and the ease of culturing makes *M. lignano *a suitable ageing model, but the major advantage is the presence of a very accessible stem cell system. The stem cells can be visualised and quantified *in vivo*, and also manipulated in several ways. Stem cells can be arrested in mitosis or in S-phase by adding colchicine or hydroxyurea respectively to the medium, or eliminated through irradiation. In contrast, the proliferation rate can also be increased by injuring the individual, which induces regeneration [11,24-28]. Furthermore, this manuscript demonstrates that it is possible to perform demographic analyses, which are necessary to draw reliable conclusions when stem cells are studied at different ages. Because the accessible stem cell population of *M. lignano *can be combined with demographic studies, this species has the potential to play a key role in obtaining a better understanding of stem cell biology in tissue homeostasis, ageing, and even rejuvenation.

## Methods

*M. lignano *is a free-living, hermaphrodite flatworm with a five-day embryonic development and a generation time of about 2–3 weeks [12]. *M. lignano *was first found in 1995 in Lignano, Italy, resulting in the first lab cultures. Afterwards, it was resampled several times, and cultures have been established in several labs. [10,11]. The species and its natural habitat is described in detail by Ladurner et al. [10,11].

In the lab, *M. lignano *is easily cultured in f/2, a nutrient-enriched artificial seawater medium at a salinity of 32‰ [29], and incubated at 20°C with a 60% relative humidity and a 13 h:11 h light: dark cycle [30]. Individuals are fed *ad libitum *with the diatom *Nitzschia curvilineata*, which is grown under identical conditions as the worms and can be obtained from the culture collection of algae (SAG) at the University of Göttingen (strain 48.91, ) [11].

Survival was followed in three replicate cultures consisting of 100 individuals each. To initiate the replicates, one-day-old juveniles were put in separate wells of a 12-well plate and, by maintaining the worms individually, reproduction and hence a mixture of individuals of different ages could be avoided. About every 30 days, individuals were put into new 12-well plates with new culture medium and diatoms to maintain *ad libitum *food resources. The number of survivors was counted about every 10 days. As in demographic studies of other model organisms [31,32], animals that died due to age-independent injury (for example rupture in *C. elegans) *were censored to ensure that the analysis reflects the natural lifespan. In *M. lignano*, age-independent death can be caused by infection with *Thraustochytrium caudivorum *[33]. This can be recognised by the characteristic dissolution of the tail plate [33]. In previous ageing cultures, it was observed at different ages (ranging from three weeks old to the oldest individuals in culture), which allowed us to conclude that it is age-independent. In the meantime, we were able to establish parasite-free cultures by optimising the working procedures and using the Triton treatment presented by Schärer et al. [33]. Kaplan-Meier survival curves were constructed (for completeness, non-censored survival curves are also given in additional file 1), and the median lifespan (50% mortality), 90^th ^percentile lifespan (90% mortality), and maximum lifespan were determined.

Characterising senescence was done by calculating the age-specific mortality rate and determining the mortality parameters of both the Gompertz and the Logistic model. The equations of these models are *y*(*t*) = A_0_e^Gt ^and  respectively [17]. Identifying which demographic model best fits the data and determining the mortality parameters was done by using WinModest software. This software uses the maximum likelihood method, which is based on the age distribution of deaths. The method provides better parameter estimates that are more consistent and less influenced by technical aspects of the experimental design such as sample size than those of other methods [17,18]. The WinModest software is very straightforward in use and is made freely available by Dr. Pletcher [17]. To determine the parameters of the Logistic model, we used the complete survival dataset of the three replicates. Because the Gompertz model describes the exponential increase in mortality rates, we used the dataset until Day 367. We chose this subset because at Day 367 the average 90^th ^percentile was reached, and after this day there is an obvious deceleration of the increasing mortality rate. The MRDT was calculated using the formula MRDT = ln(2)/G [7,20].

## Competing interests

The authors declare that they have no competing interests.

## Authors' contributions

SM designed the study, carried out the experiments, and wrote the manuscript. MW co-designed the study and participated in drafting the manuscript. PB helped to analyse and interpret the results. BPB helped to analyse the results and contributed to the manuscript drafting. GB critically revised the manuscript. All authors read, edited and approved the final manuscript.

## Supplementary Material

Additional file 1 **Uncensored survival curve of *Macrostomum lignano*.** The grey curves are the survival curves of the separate replicate cohorts. The black, bold curve represents the average overall survival curve of the three replicate cohorts. Click here for file

## References

[B1] Child CM (1911). A study of senescence and rejuvenenscence based on experiments with *Planaria dorotocephala*. Archiv fiir Entwicklungsmechanik der Organismen.

[B2] Child CM (1915). Senescence and Rejuvenescence.

[B3] Haranghy L, Balázs A (1964). Ageing and rejuvenation in planarians. Experimental Gerontology.

[B4] Lange CS (1968). A possible explanation in cellular terms of the physiological ageing of the planarian. Experimental Gerontology.

[B5] Carey JR, Liedo P, Orozco D, Vaupel JW (1992). Slowing of mortality-rates at older ages in large medfly cohorts. Science.

[B6] Valdesalici S, Cellerino A (2003). Extremely short lifespan in the annual fish *Nothobranchius furzeri*. Proceedings of the Royal Society of London Series B-Biological Sciences.

[B7] Finch CE (1990). Longevity, Senescence and the Genome.

[B8] Egger B, Ladurner P, Nimeth K, Gschwentner R, Rieger R (2006). The regeneration capacity of the flatworm *Macrostomum lignano *– on repeated regeneration, rejuvenation, and the minimal size needed for regeneration. Development Genes and Evolution.

[B9] Egger B (2008). Regeneration: Rewarding, but potentially risky. Birth Defects Research (Part C).

[B10] Ladurner P, Schärer L, Salvenmoser W, Rieger RM (2005). A new model organism among the lower Bilateria and the use of digital microscopy in taxonomy of meiobenthic Platyhelminthes: *Macrostomum lignano*, n. sp. (Rhabditophora, Macrostomorpha). Journal of Zoological Systematics and Evolutionary Research.

[B11] Ladurner P, Egger B, De Mulder K, Pfister D, Kuales G, Salvenmoser W, Schärer L, Bosch ThCG (2008). The stem cell system of the basal flatworm *Macrostomum lignano*. Stem cells: from Hydra to man.

[B12] Morris J, Nallur R, Ladurner P, Egger B, Rieger R, Hartenstein V (2004). The embryonic development of the flatworm *Macrostomum *sp. Development Genes and Evolution.

[B13] Scharer L, Sandner P, Michiels NK (2005). Trade-off between male and female allocation in the simultaneously hermaphroditic flatworm *Macrostomum *sp. Journal of Evolutionary Biology.

[B14] Mouton S, Willems M, Braeckman BP, Egger B, Ladurner P, Schärer L, Borgonie G (2009). The free-living flatworm *Macrostomum lignano*: a new model organism for ageing research. Experimental Gerontology.

[B15] Vaupel JW, Carey JR, Christensen K, Johnson TE, Yashin AI, Holm NV, Iachine IA, Kannisto V, Khazaeli AA, Liedo P, Longo VD, Zeng Y, Manton KG, Curtsinger JW (1998). Biodemographic trajectories of longevity. Science.

[B16] Curtsinger JW, Fukui HH, Townsend DR, Vaupel JW (1992). Demography of Genotypes – Failure of the Limited Life-Span Paradigm in *Drosophila melanogaster*. Science.

[B17] Pletcher SD (1999). Model fitting and hypothesis testing for age-specific mortality data. Journal of Evolutionary Biology.

[B18] Promislow DEL, Tatar M, Pletcher S, Carey JR (1999). Below threshold mortality: implications for studies in evolution, ecology and demography. Journal of Evolutionary Biology.

[B19] Yen K, Steinsaltz D, Mobbs CV (2008). Validated analysis of mortality rates demonstrates distinct genetic mechanisms that influence lifespan. Experimental Gerontology.

[B20] Arking R (1998). Biology of Aging.

[B21] Robine JM, Allard M (1998). The oldest human. Science.

[B22] Austad SN, Kristan DM (2003). Are mice calorically restricted in nature?. Aging Cell.

[B23] Altun M, Bergman E, Edstrom E, Johnson H, Ulfhake B (2007). Behavioral impairments of the aging rat. Physiology & Behavior.

[B24] Bode A, Salvenmoser W, Nimeth K, Mahlknecht M, Adamski Z, Rieger RM, Peter R, Ladurner P (2006). Immunogold-labeled S-phase neoblasts, total neoblast number, their distribution, and evidence for arrested neoblasts in *Macrostomum lignano *(Platyhelminthes, Rhabditophora). Cell and Tissue Research.

[B25] Ladurner P, Rieger R, Baguna J (2000). Spatial distribution and differentiation potential of stem cells in hatchlings and adults in the marine platyhelminth *Macrostomum *sp.: A bromodeoxyuridine analysis. Developmental Biology.

[B26] Nimeth KT, Mahlknecht M, Mezzanato A, Peter R, Rieger R, Ladurner P (2004). Stem cell dynamics during growth, feeding, and starvation in the basal flatworm *Macrostomum *sp (Platyhelminthes). Developmental Dynamics.

[B27] Pfister D, De Mulder K, Philipp I, Kuales G, Hrouda M, Eichberger P, Borgonie G, Hartenstein V, Ladurner P (2007). The exceptional stem cell system of *Macrostomum lignano*: Screening for gene expression and studying cell proliferation by hydroxyurea treatment and irradiation. Frontiers in Zoology.

[B28] Schärer L, Ladurner P, Rieger RM (2004). Bigger testes do work more: experimental evidence that testis size reflects testicular cell proliferation activity in the marine invertebrate, the free-living flatworm *Macrostomum *sp. Behavioral Ecology and Sociobiology.

[B29] Guillard R, Ryther J (1962). Studies of marine planktonic diatoms. I. *Cyclotella nana *Hustedt, and *Detonula confervacea *(Cleve) Gran. Canadian Journal of Microbiology.

[B30] Egger B, Ishida S (2005). Chromosome fission or duplication in *Macrostomum lignano *(Macrostomorpha, Plathelminthes) – remarks on chromosome numbers in 'archoophoran turbellarians'. Journal of Zoological Systematics and Evolutionary Research.

[B31] Andersen JB, Li XL, Judge CS, Zhou A, Jha BK, Shelby S, Zhou L, Silverman RH, Hassel BA (2007). Role of 2–5A-dependent RNase-L in senescence and longevity. Oncogene.

[B32] Crawford D, Libina N, Kenyon C (2007). *Caenorhabditis elegans *integrates food and reproductive signals in lifespan determination. Aging Cell.

[B33] Schärer L, Knoflach D, Vizoso DB, Rieger G, Peintner U (2007). Thraustochytrids as novel parasitic protists of marine free-living flatworms: *Thraustochytrium caudivorum *sp nov parasitizes *Macrostomum lignano*. Marine Biology.

