# Supplementary figures and images for: Demographic analysis reveals gradual senescence in the flatworm Macrostomum lignano
Source: Front Zool. 2009 Jul 30;6:15. doi: 10.1186/1742-9994-6-15 (PMC2724480; doi:10.1186/1742-9994-6-15)

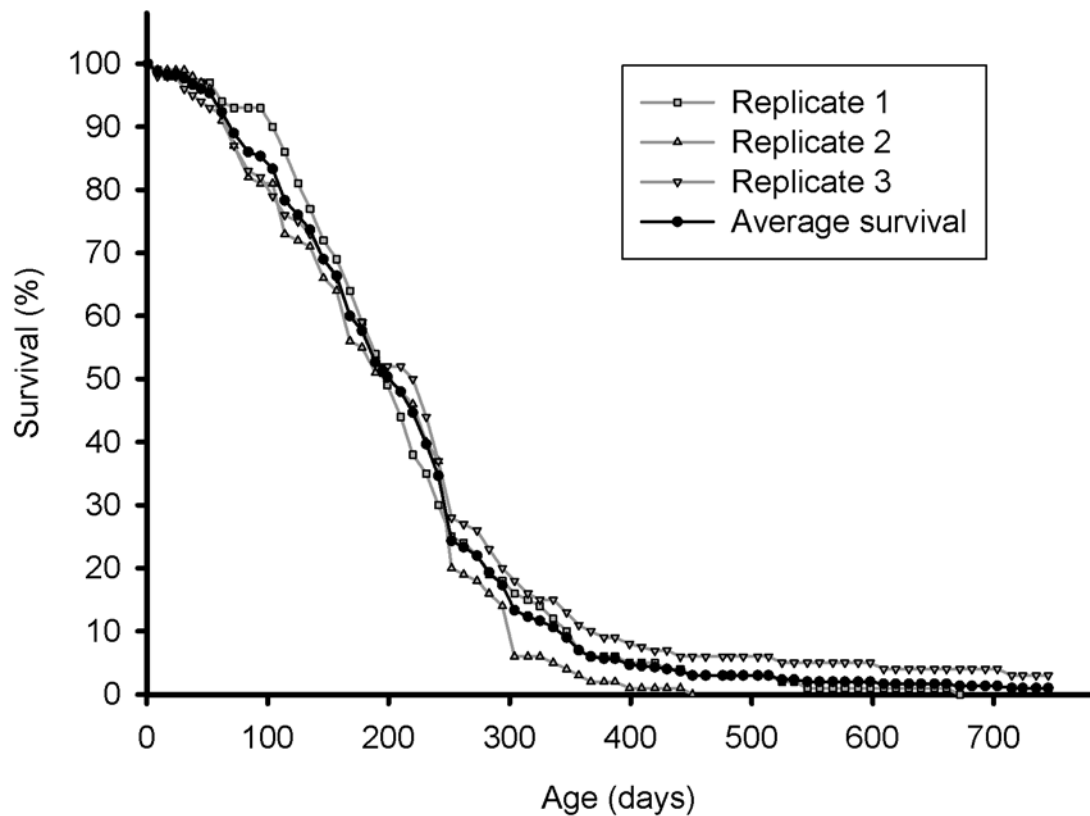

Supplement: Additional file 1 — Uncensored survival curve of Macrostomum lignano. The grey curves are the survival curves of the separate replicate cohorts. The black, bold curve represents the average overall survival curve of the three replicate cohorts. [file 1742-9994-6-15-S1.pdf]
